# The terpenes of leaves, pollen, and nectar of thyme (*Thymus vulgaris*) inhibit growth of bee disease-associated microbes

**DOI:** 10.1038/s41598-018-32849-6

**Published:** 2018-10-02

**Authors:** Natalie Wiese, Juliane Fischer, Jenifer Heidler, Oleg Lewkowski, Jörg Degenhardt, Silvio Erler

**Affiliations:** 1 Institute of Pharmacy, Pharmaceutical Biotechnology, Martin-Luther-University Halle-Wittenberg, Hoher Weg 8, 06120 Halle, Saale Germany; 2 Institute of Biology, Molecular Ecology, Martin-Luther-University Halle-Wittenberg, Hoher Weg 4, 06120 Halle, Saale Germany

## Abstract

Honey bees are highly prone to infectious diseases, causing colony losses in the worst case. However, they combat diseases through a combination of their innate immune system and social defence behaviours like foraging for health-enhancing plant products (e.g. nectar, pollen and resin). Plant secondary metabolites are not only highly active against bacteria and fungi, they might even enhance selective foraging and feeding decisions in the colony. Here, we tested six major plant terpenes and their corresponding acetates, characterizing six natural *Thymus vulgaris* chemotypes, for their antimicrobial activity on bacteria associated with European foulbrood. Comparison of the inhibitory activity revealed the highest activity for carvacrol and thymol whereas the acetates mostly did not inhibit bacterial growth. All terpenes and acetates are present in the nectar and pollen of thyme, with pollen containing concentrations higher by several orders of magnitude. The physiological response was tested on forager and freshly emerged bees by means of antennal electroantennography. Both responded much stronger to geraniol and trans-sabinene hydrate compared to carvacrol and thymol. In conclusion, bee-forageable thyme product terpenes (mainly from pollen) yield effective antibiotic activity by reducing the growth of bee disease-associated bacteria and can be detected with different response levels by the honey bees’ antennae. This is a further step forward in understanding the complex pathogen-pollinator-plant network.

## Introduction

Bees of the subfamily Apinae (mainly *Apis mellifera*, *Bombus* sp.) are of major economic and agricultural importance for human nutrition and bee product business^1^, but also for the pollination service of wild plants to facilitate plant reproduction and maintain the natural food chain^2^. Within the complex network of plants and their pollinators, parasites and pathogens are becoming increasingly prominent in affecting this interaction by harming pollinators^3^. They can be easily transmitted within the colony, between colonies or hives and different species which forage on the same flowers^4^. Once in the colony, constant temperature, humidity and food supply offer excellent conditions for growth and reproduction of the pathogen^3^. However, bees are not defenceless against parasites and diseases. In addition to their innate immune system they developed several behavioural defence mechanisms, called ‘social immunity’^5^, including foraging and storage of highly antibiotic plant products. Nectar, pollen and resin (propolis) are used for self-medication on the colony- and individual level^6^. Plant nectar, which is processed by honey bees to storable honey, is particularly full of secondary metabolites and the self-medication potential of honey (inhibiting pathogen growth and/or reducing pathogen loads) has been shown repeatedly^6–8^. Specific compounds of floral nectar (e.g. anabasine, nicotine and thymol) can also reduce parasite loads in bumble bees^9^. The central active compounds of foraged plant products are volatile (essential oils) and non-volatile plant secondary metabolites that most probably have evolved to prevent nectar spoilage, attract pollinators or deter nectar robbers^10^. Alternatively plant secondary metabolites are a pleiotropic consequence of plant defence against herbivores^11^.

Essential oils are hydrophobic, volatile plant compounds, mostly isolated by means of hydro-/steam-distillation. These oils and their single compounds extracted from cinnamon, lavender, oregano, rosemary, sage, thyme and many more, were the first plant extracts recorded for their antibiotic activity on human pathogens and their application in ethnopharmacology^12,13^. Above all, some thyme species were used to discover the antibiotic potential of essential oils on honey bee parasites and pathogens; e.g. Andean thyme (*Acantholippia seriphioides*) against the bacterium *Paenibacillus larvae*;^14^ *Thymus satureoides*, *T*. *serpyllum* and *T*. *vulgaris* essential oils against the fungus *Ascosphaera apis*^15^. However, most of the studies used essential oils or plant extracts originating from common or garden thyme (*Thymus vulgaris*), showing antibacterial (*P*. *larvae*^16,17^), antifungal (*A*. *apis*^18^, *Aspergillus flavus* and *Aspergillus niger*^19^), and acaricide (*Varroa destructor*^20–22^) activity. Only a handful of studies tested the antimicrobial activity of plant secondary metabolites (of floral and bryophyte origin, incl. tea tree) not only against *P*. *larvae* but also against *Melissococcus plutonius* (both causing severe brood diseases in honey bees) with sometimes very weak activity^23–27^. *M*. *plutonius* causes the honey bee brood disease European foulbrood (EFB)^28^. EFB is a severe brood disease causing colony loss in parts of Europe^28^. *M*. *plutonius* infects young larvae via ingestion of contaminated food (for a review on disease ecology and pathogenesis see Forsgren^28^). The gastrointestinal tract, mainly the gut, is not only the area through which larvae get infected, it is also the site where bacteria might be exposed to the phytochemicals that are added to the larval food. Disease-associated bacteria (*Bacillus pumilus*, *Brevibacillus laterosporus*, *Enterococcus faecalis*, *Paenibacillus alvei* and *Paenibacillus dendritiformis*), the so-called secondary invader, can be found in chronically diseased colonies and larval remains, and some of them have been attributed to increased disease symptoms and infection success^7,28,29^. However, their function for the course of the disease is controversially discussed^28,30^.

Nowadays, more than 360 different essential oil volatile components of 162 taxa of the genus *Thymus* have been chemically characterised, with more than 90% of the total oil content being composed of monoterpenes, among which thymol and carvacrol are the most abundant^31^. The chemical composition of thyme essential oils varies vastly between species^32^ and even within species. For *T*. *vulgaris* six different chemotypes are known: thymol (T-type), carvacrol (C-type), geraniol (G-type), linalool (L-type), α-terpineol (A-type) and trans-sabinene hydrate (U-type)^33^. These chemotypes are characterised by their dominant monoterpene alcohol which provides more than 50% of the respective essential oil. In addition to the major monoterpenes, other characteristic compounds can be found in each of the chemotypes. These are the corresponding acetates of the monoterpenes (geranyl acetate in the G-type; α-terpinyl acetate in the A-type and linalyl acetate in the L-type). So far, only a single study tested the antimicrobial potential of some chemotype essential oils, without any profound analysis on the origin of variance between chemotypes^34^.

For most of the thyme essential oil single compounds, except for thymol and carvacrol, antibiotic activities are not well characterised. For thymol, antimicrobial activities were tested with *P*. *alvei*, *P*. *larvae*^35,36^, antifungal activities were tested with *A*. *apis*, *Nosema* sp., *N*. *ceranae*^37–39^, and acaricide activities were tested with *Acarapis woodi*, *V*. *destructor*^38,40,41^. For carvacrol, activities were reported with *A*. *woodi*^41^ and *P*. *larvae*^42^. For geraniol, linalool and linalyl acetate which are often constituents of thyme essential oils, repellent activity on *Varroa* has been mentioned^21^. For controlling *V*. *destructor*, some plant compound based commercial formulations are nowadays used by the beekeeping industry (Apiguard^®^, ApiLife Var^®^ and Thymovar^®^) with thymol as the active ingredient. In-hive, not only the antibiotic activity of secondary compounds collected by foraging, but also their activity in stored honey, are believed to be of high relevance in terms of colony medication via foraging for health. However, only a few studies tested the effectivity of thyme honey (e.g. Greek and Turkish origin) on Gram-positive and Gram-negative bacteria, by reaching minimum inhibitory concentrations of 6–13% honey^43,44^.

Nevertheless, for most of the monoterpenes and their corresponding acetates, no specific function has been reported regarding herbivore defence, pollinator attraction, nectar conservation or antibiotic activity for the host organism. Here, we want to quantify the amount of each compound in nectar and pollen of several *T*. *vulgaris* chemotypes, and test and compare the antimicrobial potential of all six major monoterpenes and the three acetates on the European foulbrood causing agent *M*. *plutonius* and disease-associated bacteria. If plant secondary metabolites have a central role for plant-pathogen-pollinator interaction, at least one of the two plant products need to exhibit terpenes at concentrations relevant to, at least partially, inhibit bacterial growth. In the last step, foraging bees have to perceive the different plant terpenes for a potential selective foraging decision. Differences in olfactory responses in newly emerged worker and forager honey bees were used to assess whether bees can discriminate between different substance classes and chemical modifications of single substances. By combining compound quantification, estimation of their antibiotic potential and putative impact on foraging, this study gives an insight into plant-pathogen-pollinator interactions and the role of plant secondary metabolites, which might be highly important for the evolution of social immunity.

## Results

### Concentrations of *T. vulgaris* terpenes in nectar, pollen and leaves

Nectar and pollen terpene content differed from each other by more than two orders of magnitude, with pollen showing highest concentrations (Table 1). Carvacrol, thymol, geraniol, linalool, α-terpineol and trans-sabinene hydrate were detectable at 0.24 to 7.67 ppm in nectar samples collected from distinct chemotypes (Table 1). Among the acetates, only α-terpinyl acetate was present in nectar with 1.69 ppm. Data for pollen samples were only available for the A-, G-, and L-chemotype with values ranging from 17.11 ppm for geranyl acetate (G-type) to 742.06 ppm for linalool (L-type). The ratio between major terpene and the corresponding acetate differed from 2.5-times (G-type) to 8.5-times (L-type), with the exception that the acetate was more present (4.2-times) than the non-acetate for the A-type pollen (Table 1).

**Table 1:** Terpene concentrations (ppm) in nectar, pollen (anthers containing microsporangia) and leaf samples of six different *T*. *vulgaris* chemotypes. Concentrations are given by fresh mass.

Chemotype	Substance	Nectar			Pollen			Leaves		
		mean	±SD	replicates	mean	±SD	replicates	mean	±SD	replicates
C	Carvacrol	0.24	0.03	2	n.a.			6579.48	1188.65	4
T	Thymol	7.67	0.03	3	n.a.			14209.81	856.03	3
G	Geraniol	0.99	0.21	3	43.08		1	4873.78	685.13	3
	Geranyl acetate	n.d.			17.11		1	3855.82	259.23	3
A	α-Terpineol	2.83	0.55	3	57.97	3.87	3	3765.24	544.53	3
	α-Terpinyl acetate	1.69	0.36	3	243.37	14.30	3	17624.17	1503.91	3
L	Linalool	2.18		1	742.06	84.22	3	18067.50	2919.12	3
	Linalyl acetate	n.d.			87.64	12.30	3	2195.51	371.80	3
U	trans-Sabinene hydrate	2.45	0.23	3	n.a.			7349.48	2445.48	3

SD: standard deviation; n.d.: not detected; n.a.: no pollen samples available, only female flowers from this chemotype.

The terpene concentrations in leaf extracts were several orders of magnitude higher than in pollen or nectar, varying from approx. 2,000 to 20,000 ppm (Table 1). Ratios of terpenes and acetates showed the same trends as observed for the pollen samples with 1.2- to 8.3-times differences.

### Antimicrobial activity of *T. vulgaris* terpenes

**Figure 1:**
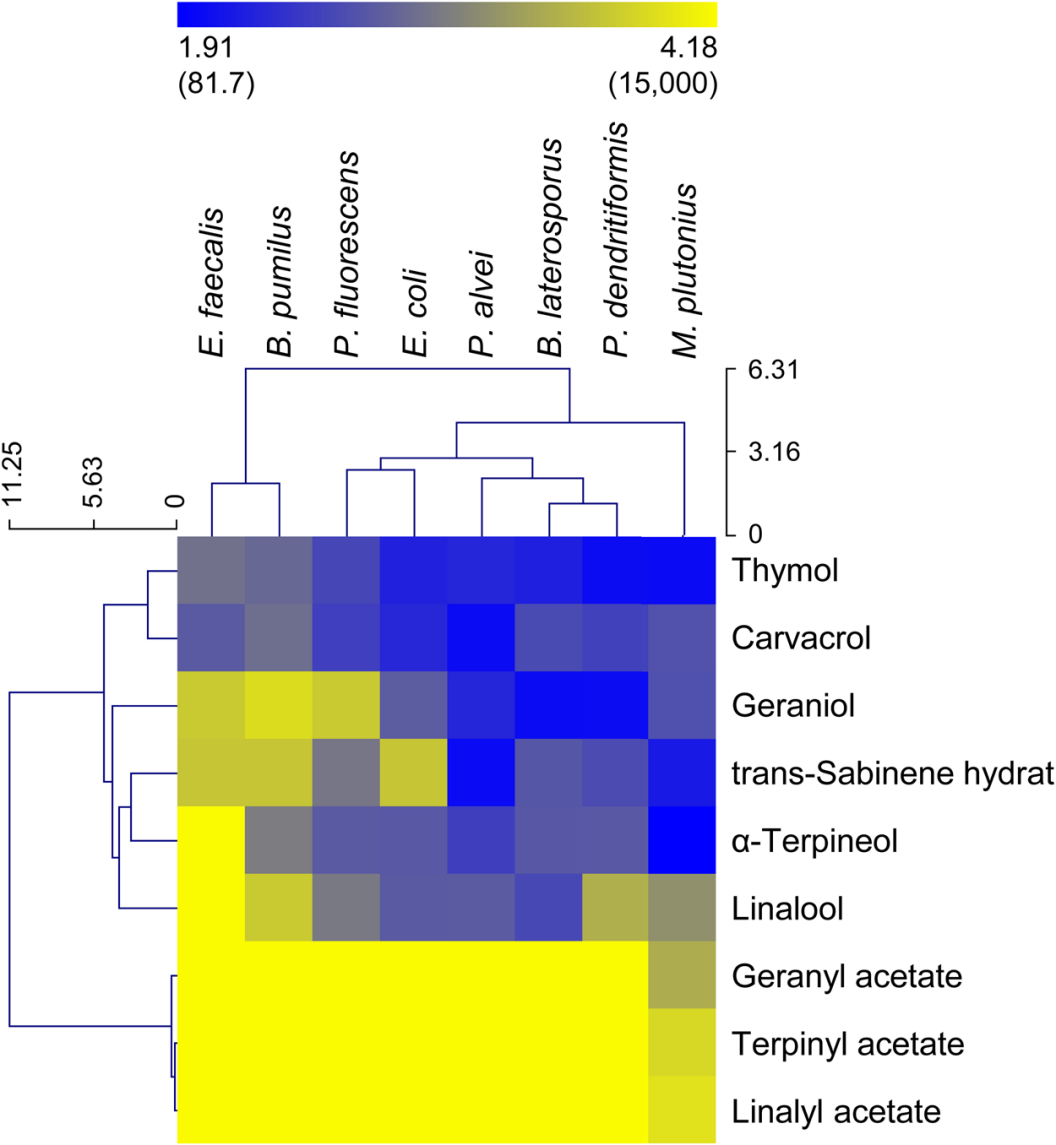
Antimicrobial activity of *T*. *vulgaris* major essential oil terpenes. Activities are shown as IC_50_ (relative half maximum inhibitory concentration, for actual inhibitory values see Tables S1) values (log-transformed scale and non-transformed ppm values in brackets) for *M*. *plutonius*, European foulbrood associated bacteria (*B*. *laterosporus*, *B*. *pumilus*, *E*. *faecalis*, *P*. *alvei* and *P*. *dendritiformis*) and two non-bee associated bacteria (*E*. *coli* and *P*. *fluorescens*). Values define the growth response based on growth rate and are based on 5 replicates for each species and compound. Light yellow means the substance tested showed no inhibitory effect on bacterial growth. Branch lengths in dendrograms (for bacteria and substances) produced from hierarchical cluster analysis (Manhattan distance, average linkage clustering) correspond to relative degree of similarity between branches.

All terpenes, except for the acetates, showed highly variable antimicrobial activity towards the bacterial species tested (Fig. 1, Supplementary Table S1, Supplementary Data S1). At the highest concentration tested (15,000 ppm), no growth inhibitory effect was observed for geranyl-, linalyl- and α-terpinyl acetate, except for the decelerated growing *M*. *plutonius*, forming a separate group in Fig. 1. On the contrary, carvacrol and thymol most successfully reduced the growth of all species with low effective concentrations (IC_50_ < 1000 ppm, in 75% of the cases even <500 ppm). The effectivity of the terpenes geraniol, linalool, α-terpineol and trans-sabinene hydrate, ranges between highly active (IC_50_ values of ~100–500 ppm) and no inhibition, depending on the bacterial species (Fig. 1, Supplementary Table S1).

*B*. *laterosporus*, *P*. *alvei*, *P*. *dendritiformis* and *M*. *plutonius* were the most sensitive bacteria (IC_50_ mostly <1000 ppm, even <500 ppm in more than 75% of the cases) (Fig. 1). Comparable response patterns at low sensitivity were observed for the Gram-negative bacteria *E*. *coli* and *P*. *fluorescens*. *B*. *pumilus* and *E*. *faecalis* formed an additional cluster in Fig. 1 due to their high terpene resistance, in particular against geraniol, linalool, α-terpineol and trans-sabinene hydrate.

In comparison to their respective controls (medium only), all bacteria species showed delays of up to ~20 h for growth in presence of *T*. *vulgaris* terpenes, as determined by the comparison of starting points of logarithmic (exponential) growth (Supplementary Table S2). A potential delay was not estimated for *M*. *plutonius* due to its demanding cultivation, even under optimized conditions.

The estimation of bacterial growth for *B*. *pumilus*, *E*. *faecalis*, *E*. *coli* and *P*. *fluorescens* in the presence of trans-sabinene hydrate became difficult, as the substance was insoluble at concentrations of 5000 (only for *P*. *fluorescens*), 10000 and 15000 ppm in the tested media (Supplementary Tables S1, S2).

*M*. *plutonius* was the only strain which was not resistant to the acetates. For the non-acetates this species displayed comparable IC_50_ values as seen for the other bacteria (Fig. 1, Supplementary Table S1). The acetate-sensitivity might be a cultivation effect (e.g. variance in cultivation time: 5 days *vs*. 24 h, environment: CO_2_-incubator *vs*. aerobe and shaking). Nevertheless, a typical *M*. *plutonius* growth pattern was observed for all substances, without any anomalies, and a constant growth rate (generation time) of 4.5 h ± 0.51 h (mean ± SD) was estimated. Further, *M*. *plutonius* grew faster (within 24–48 h) in medium containing 1–2% DMSO. DMSO may be used as an alternative electron acceptor for anaerobic respiration of *M*. *plutonius*^45^.

### Antennal response towards terpene exposure

**Figure 2:**
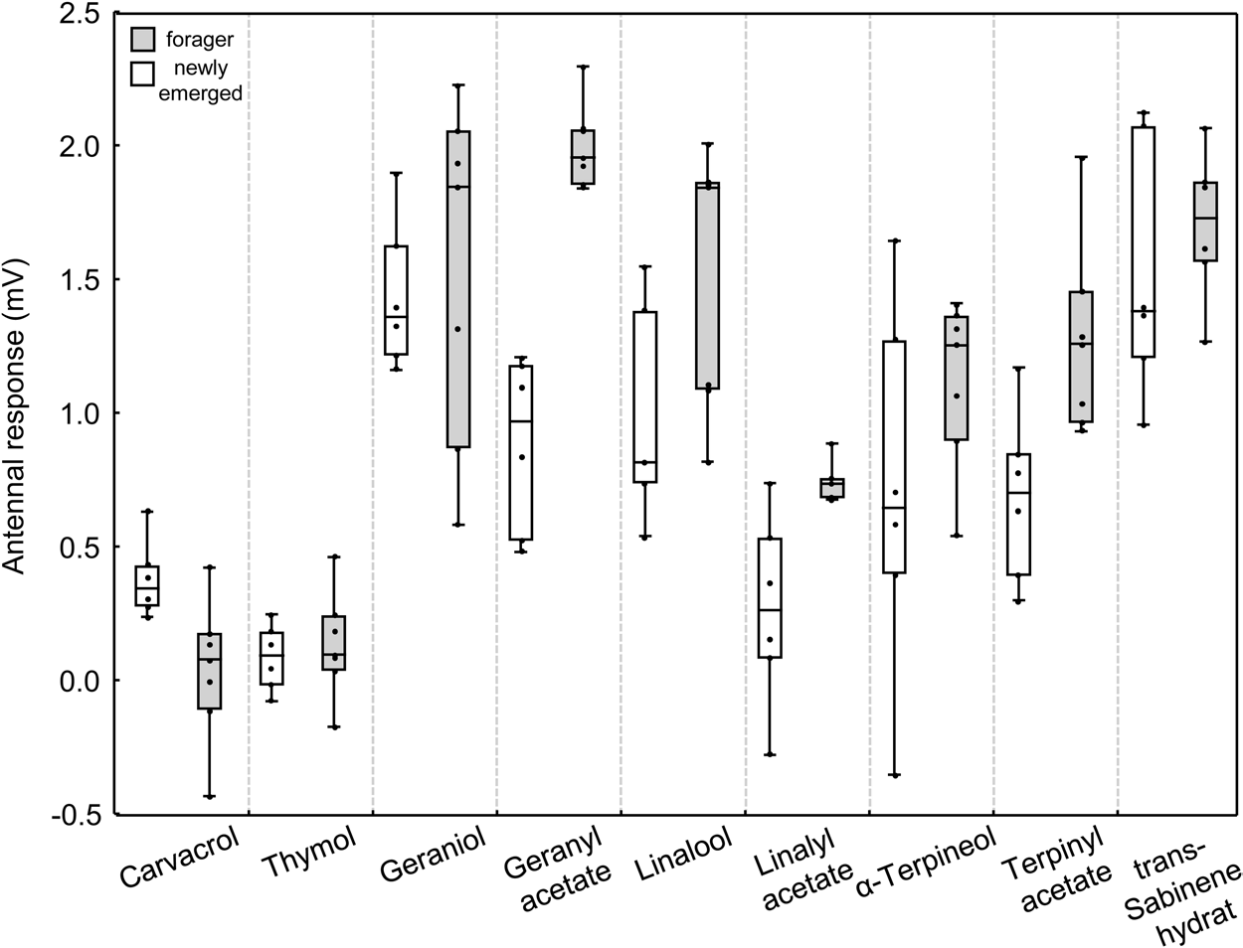
Antennal response of newly emerged worker (white boxes, n = 6 bees) and forager (grey boxes, n = 7 bees) honey bees to six *T*. *vulgaris* terpenes including their acetates. Shown are *medians* with 25–75% (*box*) and min-max range (*whisker*). (Black dots are single bee data points, representing a single antenna tested per substance).

The EAG responses differed strongly between the two different groups of bees (GLM: *F*_1,114_ = 25.17, *P* < 0.0001) and the substances tested (GLM: *F*_8,114_ = 29.22, *P* < 0.0001). Newly emerged bees responded more weakly overall to substances than the forager bees (Fig. 2). The only difference between both (newly emerged *vs*. forager), on the level of single substance comparison, was observed for geranyl acetate where forager bees responded much more strongly than the others (Bonferroni post-hoc test, *P* = 0.0001) (Fig. 2).

Among the tested substances, the EAG responses can be clustered in three major groups: (1) low response (mean ± SD: 0.16 ± 0.23 mV, min-max: −0.43–0.64 mV) - carvacrol and thymol, (2) medium response (0.80 ± 0.52 mV, −0.35–1.97 mV) - linalyl acetate, α-terpineol, and α-terpinyl acetate, and (3) high response (1.47 ± 0.51 mV, 0.49–2.31 mV) - geraniol, geranyl acetate, linalool, trans-sabinene hydrate (Fig. 2, Supplementary Data S1). No differences were observed for the response to acetate and non-acetate pairs for the forager or newly emerged bees (post-hoc multiple comparisons following Kruskal-Wallis ANOVAs, *P* > 0.05).

## Discussion

One aspect of this study was the quantification of nectar and pollen terpenes as representative of plant secondary metabolites that may have a central role in plant-pathogen-pollinator interaction with bees foraging for biologically active plant products^10,11^. Quantification revealed that terpene concentration varies by several orders of magnitude for nectar and pollen samples of the different *T*. *vulgaris* chemotypes. However, in-hive concentrations might be lower or possibly higher due to the bees’ handling of nectar and pollen. Once the forager bees collect nectar and offered it to the in-hive bees, nectar will be modified (mostly an increase in viscosity and the addition of bee enzymes) during the process of ripening and finally stored as honey^46^. This process will significantly affect the final concentration of nectar volatiles (essential oils) in the stored product, which has a highly reduced water content (approx. 19% or less) in comparison to nectar^47^. On the one hand, ripening and thickening will concentrate nectar metabolites, but on the other hand, highly volatile essential oils might evaporate by increased fanning activity before cell sealing^47^. The complete process of honey ripening^46^, especially substance in- or decreasing is not well investigated *per se*, and in particular not for thyme nectar. Only a few studies estimated terpene content of thyme honey and found values in concentrations that are known to reduce pathogen loads^9^ (thymol: 0.27 ppm for thyme honey, 0.12–0.36 ppm for non-thyme honeys)^48^ or with very low values: carvacrol (0.9%), linalool (0.1%), α-terpineol (0.1%), and thymol (0.1%)^49^. More informative values, not only given in percentage against the total peak area^49^, also showed very low concentrations for carvacrol (max. 0.017 ppm), linalool (max. 0.12 ppm), α-terpineol (max. 0.043 ppm), and thymol (max. 0.018 ppm) for different honeys including thyme honey^50^. A plausible explanation for the high variance are differences in storage conditions (e.g. storage time, temperature, or light) by the beekeeper, time of honey harvest and delay of subsequent compound analysis. If honey is not harvested freshly after being collected by the bees and stored in a freezer, volatiles will begin to evaporate and can consequently be detected only in very low quantities.

In comparison to honey, nectar terpene concentrations are usually measured from fresh material and give a more precise picture of nectar quality. For the six different chemotypes analysed in this study, terpene concentrations do not differ much for each substance and are potentially characteristic for each chemotype. The thymol concentration (7.67 ppm), at least, is well in the range of a previous study (2.22–8.2 ppm)^51^, even though a different extraction method and plant material (cultivars) were used. For pollen, more data are needed to conclude if the detected values are representative for the distinct chemotypes. The thymol content of pollen could not be determined in this study since no male flowers were available. Nevertheless, data from hive pollen samples for the U.S.A. National Survey showed that thymol concentrations can vary from 0.0263 to 55.8 ppm^52^, which is in the range of the other pollen terpenes analysed in the current study. Apart from that, the higher values may have resulted from sampling pollen from *Varroa*-treated hives (by fumigation, see introduction). Altogether we assume that *T*. *vulgaris* nectar and especially its pollen contain terpene concentration with a potential of activity against parasites and pathogens if stored or directly distributed and consumed in the hive. The concentration of leaf volatiles are orders of magnitude higher than for nectar and honey, and may not contribute directly to the health status of honey bee colonies. However, leaf-cutter bees (*Megachile rotundata*) harvesting plant leaves to coat their brood chambers may benefit from the amount of leaf terpenes protecting their brood against, for instance, chalkbrood^53^. Whether and how thyme leaves are used by bees is not completely understood, but the high terpene concentrations may contribute to orientation and decision making during foraging flights^54^.

For estimating the antibiotic potential of the selected plant terpenes, bacterial growth inhibition was measured for several bee-associated bacteria. For some of these bacteria species (e.g. *E*. *faecalis*) it is known that herb essential oil mixes are biologically much more effective than single compounds (e.g. carvacrol, thymol, geraniol, linalool, α-terpineol, geranyl acetate)^13^. Here, we have shown that carvacrol and thymol performed best in inhibiting bacterial growth in comparison to the other plant terpenes tested. For both substances low concentrations were sufficient to inhibit bacterial growth by 50%. In some cases (MIC - minimum inhibitory concentration, showing the first sign of growth inhibition^55^: 100–300 ppm for *P*. *alvei*, *P*. *dendritiformis*, *E*. *coli*, this study) they showed comparable activity to the complete essential oil bouquet (MIC: 100–200 ppm^16,17^). This might be due to their phenolic structure, as all other thyme terpenes tested in this study are non-phenolic (Fig. S2). Chemical modifications of terpenes strongly affect their antibiotic activities. For example, acetylation led to the complete inactivation of geraniol, linalool and α-terpineol in the current study (Fig. 1), except for their activity against *M*. *plutonius*. This inactivation not only causes reduced or even absent antibiotic activity against bacteria but also towards fungi^13,56^. Hence, thyme nectar terpenes vary strongly in their antibiotic activity and this variance is defined in part by their modifications. Such a relationship between chemical structure (acyl group size) and antibiotic activity against bacteria (*P*. *larvae*) and fungi (*A*. *apis*) was recently discovered for propolis dihydroflavonols^57^. Among the foraged and self-produced bee products with high effectivity on bee pathogens^6^, propolis is the hive product for which the raw material and its compounds were studied in most detail, for instance regarding its effect on *P*. *alvei*^58^. Future studies have to determine the effectivity of *T*. *vulgaris* plant nectar and pollen terpenes in larval food to cure larval infectious diseases like American or European foulbrood. However, first it is mandatory to estimate how much terpenes can be detected in larval food under normal hive conditions, as variance between IC_50_ values and concentration in nectar and pollen is quite high. The high amounts needed to inhibit bacteria *in vitro* may only be reached by feeding pollen (to drone and worker brood) or by accumulating terpenes while producing honey. On the other hand *in vitro* IC_50_ values are not necessarily the same for *in vivo* studies. Lower concentrations might be sufficient as synergistic effects or additional antibiotic activity by other substances of the larval food cannot be excluded^59^. This means for some terpene concentrations of nectar and mainly pollen a sufficient amount is present to reduce pathogen growth *in vitro*, for most of the bacteria. A total inhibition, in particular *in vivo*, may only be reached by combinations of terpenes, other plant metabolites and hosts’ own proteins and enzymes. By measuring nectar and pollen terpene concentrations, and the antibiotic activity of single plant terpenes, the direct significance for self-mediation has not been tested here and has to be evaluated in future studies.

From the beekeepers’ biosecurity perspective, nectar, pollen or honey terpenes not only have to be highly specific and efficient in their activity against parasites and pathogens, they also have to be safe for the host organism at concentrations applied for pathogen treatment. In all bacteria-terpene combinations, the observed antimicrobial activity (IC_50_ values) of the six active terpenes was far below the well documented toxic concentration of *T*. *vulgaris* essential oils for adult honey bees (>10000 ppm within 48 h)^60^. Grobov and colleagues^38^ reported a LD_50_ of 8900 ppm for thyme essential oils ingestion. Both studies cannot be compared due to the lack of details in experimental set-up including exposure time and age of the tested bees. For individual compounds, toxicity data are available only for thymol. It was shown to be not toxic at the tested concentrations towards adult bees in cage experiments^61,62^, whereas larval weight and pupal survival were reduced after six days of feeding with 500 ppm^63^. Short term thymol exposure (48 h, 2^nd^ instar larvae, acute toxicity) led to a LD_50_ value of 2010 ppm, while chronic toxicity was estimated with 703 ppm (LD_50_, 5^th^-instar larvae) after six days of permanent feeding^63^. However, larval stages differ between short term and chronic toxicity. From these previous studies we can conclude that carvacrol and thymol can be tested *in vivo* for their self-medication potential on honey bee larvae without harming the host, whereas more detailed toxicity studies are needed for geraniol, linalool, α-terpineol and trans-sabinene hydrate. Further, it should be noted that LD_50_ values for short term application are usually much higher than for chronic exposure. This means that the higher antibiotic activity values for geraniol, linalool, α-terpineol and trans-sabinene hydrate are theoretically usable for the short term without harming bee larvae.

Acute and chronic terpene consumption not only influences toxicity for the host but also effectivity against its parasites and pathogens. Chronic thymol feeding (0.2 ppm for 10 days) had no effect on the trypanosome *Lotmaria passim*, the microsporidium *Nosema ceranae* and Deformed Wing Virus (DWV), though a single dose reduced DWV loads^62^. Exposure time, application type (fumigation, liquid or solid food) and concentration range (with number of dilution steps) are the critical parameters for estimating LD_50_ doses and effects on parasites and pathogens, as in some cases contradictory observations can be found. From bumblebees and the trypanosome gut parasite *Crithidia bombi* we know that low concentrations of thymol can reduce disease loads of infected bees (0.2 ppm)^9^ or have no effect with even higher concentrations (0.2–2 ppm)^64^. Testing different parasite strains revealed a more than 4-fold variance of *C*. *bombi* phytochemical resistance (EC_50_ values: 4.5–22 ppm)^51^. This shows that not only must the above mentioned criteria be taken into account for estimating antibiotic activities, but also host genotype, age, and parasite strain. Lastly, a recent study showed that repeated experiments led to variable EC_50_ values (5–50 ppm)^65^. Differences of such orders of magnitude for effective doses seem to be realistic simply by switching between *in vivo* to *in vitro*, as nicely shown for the bumblebee *B*. *impatiens* and its parasite *C*. *bombi*^65^. As indicated above, *in vivo* studies are urgently needed for bacterial bee diseases and brood diseases. Most of the aforementioned facts result from adult bee infections with viruses or eukaryotes that live intracellularly or in the gut system. The trypanosomes (e.g. *Crithidia*, *Lotmaria*) are single cell eukaryote organisms attached to the gut epithelium, whereas bacteria like *M*. *plutonius* are single cell prokaryotes that also live in the gut but might cluster together to better survive the hosts’ defence system^66^. Results from different groups of parasites and pathogens are currently not comparable for generating any general conclusions regarding plant metabolite health enhancing effects.

Nectar and pollen essential oils are always a mix of several terpenes and they might interact with each other causing additive, synergistic or antagonistic effects. Several studies proved synergistic effects for thymol and nicotine^64^ or thymol and eugenol against *C*. *bombi* with high variance across parasite strains^65^. The same was observed for bacteria with synergistic effects of thymol, carvacrol, eugenol and cinnamaldehyde against *E*. *coli*^67^ and thymol, carvacrol and linalool acting partly synergistically against *Klebsiella pneumoniae*^68^. However, it is noteworthy that the effectivity of essential oil compound mixtures strongly depends on the tested bacteria species and strain, as well as on assay conditions. The very same mix can act additively, synergistically, or antagonistically, as recently summarised by Bassolé and Juliani^69^.

The mechanisms of plant essential oils to reduce growth or actively kill bacteria and fungi are only partly known^70^. Thymol is involved in the alteration of the cell membrane and the cell wall of yeast which leads to its antifungal activity^71^. Similar and even more complex mechanisms can be seen for the effect of thymol and linalyl acetate on bacteria. These terpenes damage or destroy the bacterial cell membrane and cell wall^72^, resulting in alterations of membrane permeability and leakage of intracellular material^73^. Comparing their efficiency in destroying large unilamellar vesicles (liposomes), however, revealed that linalyl acetate has a much lower capability for disrupting lipid membranes than thymol^73^. Carvacrol and thymol treatment also led to ATP leakage following cytoplasmic membrane disruption^74^, increase of the permeability of cells, dissipation of pH gradients and leakage of inorganic ions^75^.

Nectar and pollen are resources for antibiotic plant terpenes. However, it is not clear if and how bees respond differently to different thyme terpenes and how chemical modification (acetylation) might modify antennal response. This specific response might be of high relevance for the colonies’ foraging decision and the larvae feeding decision, realised on the single individual level. From previous studies it is know that during foraging flights honey bees can discriminate between odour blends of leaves and flowers of different plant chemotypes, for example the carvacrol or thymol chemotype of *Majorana syriaca*^76^, which might help decision making for effective foraging. Unlike flowers of different chemotypes, flowers of the same cultivar cannot be separated. Only the floral scent of different cultivars can be discerned as a function of intensity and chemical distance among scents^77^. Furthermore, the same authors suggest that honey bees are capable of using all floral volatiles tested to discriminate variances in scent^77^.

Forager bees perceive, recognize, and identify olfactory signals and are able to transfer this information to other bees during forager recruitment^76^. However, for most plant species we are lacking experimental evidence for whether bees use the whole chemical bouquet or only single key compounds to discriminate between chemotypes. Moreover, we have to ask if honey bees can detect all substances characteristic of plant-specific floral nectar or pollen to make a foraging decision. Laboratory experiments already showed that bees can learn to discriminate very similar odours^78^. They can also discriminate a higher or lower concentration of a particular odor^78^. How much time a bee needs to make such a decision is independent of the difficulty of the discriminatory task, and decision time is quite constant for different odorant pairs^78^. Learning studies also showed that there is some variance in odorant discrimination depending on substances. In the case of similar compound mixes proportions of substances have to be relatively dissimilar^79^. Altogether, these results have important implications for olfactory processing models^78^.

In this study, we could show that newly emerged workers and foragers might discriminate between the different characteristic *T*. *vulgaris* terpenes, despite some of them being structurally similar (like carvacrol and thymol). This might also mean that bees (forager) can discriminate between chemotypes and stored nectar/honey types (nurses). The overall EAG responses were much lower for the newly emerged workers than for the forager bees, but response patterns for the different substances were similar for both. This result is consistent with previous studies showing that age and rearing conditions significantly influence antennal response. Right after hatching bees need about 4 days for maturation of EAG responses and show a plastic response to pure and complex odours from the 15^th^ day^80^. In this study, we could show that the antennal response of both revealed three distinct response clusters from low to high, with very low response for the phenolic substances (carvacrol and thymol). Geraniol and geranyl acetate elicited the highest response, which is not surprising as geraniol is a major compound of the Nasanov/aggregation pheromone of honey bees^81^ and should therefore be detected with highest sensitivity. Over all substances tested, we measured no difference between the major thyme terpenes (like geraniol) and their acetylated forms (e.g. geranyl acetate). The different response groups and low variance for the different substances might be explained by several mutually non-exclusive theories, based on the interaction of honey bees’ odorant-binding proteins (n = 177), odorant receptors (n = 21) and odorant-degrading enzymes^82,83^:

1. Odorant-binding proteins and/or odorant receptors of the bees’ antennae may primarily discriminate between phenolic and non-phenolic substances, as they bind a wide range of odorant molecules.
2. Receptor density and/or saturation for phenolic substances is much lower than for non-phenolic substances, leading to lower antennal response.
3. Odorant-binding proteins do not discriminate between acetylated and non-acetylated substances as they mainly recognize specific motifs of certain molecules.

Honey bees are assumed to be able to discriminate between different floral terpenes and variable concentrations. For *T*. *vulgaris* essential oils it is known that bees can discriminate different concentrations and change their feeding behaviour accordingly^60^. However, all thyme-specific chemical communication and signal transmission steps from the bees’ antennae to the brain, as well as downstream neuronal signalling, leading to the behavioural response of the bee, are currently a huge black box and have to be investigated in future studies. Recently, Bonnafé and colleagues^84^ made the first step towards understanding the molecular processes initiated by topical application of thyme terpenes. Within the honey bees’ brain, thymol exposure rapidly up- and down-regulated genes coding cellular targets (e.g. octopamine receptor OA1 and transient receptor potential-like channel). The same authors claimed that thymol exposure may modify foraging capabilities by altering memory and learning^84^.

In conclusion, thyme (*T*. *vulgaris*) nectar and pollen terpenes, in particular thymol and carvacrol, are suitable antibiotic plant secondary metabolites with variable efficiency in inhibiting the growth of Gram-positive (bee pathogens) and Gram-negative bacteria. The acetylation of several terpenes resulted in nearly complete inactivation of their antibacterial activity (e.g. geraniol, linalool, and α-terpineol). What it means exactly for pollinator-plant interactions is currently unknown. Determination of compound quantities in nectar and pollen revealed that for some substances the terpene concentration, detected in pollen, is sufficient to reduce pathogen growth, although not fully for most of the bacteria. Forager bees are able to discriminate between different terpenes, though with a lower response to phenolic than to non-phenolic substances. This indicates that plant secondary metabolites may be relevant for prophylactic and therapeutic medication of managed honey bees to cure harming diseases with natural compounds. However, this has not been addressed here directly and must be tested in future studies, as the used terpenes may act as honey bee attractant or repellent.

Consequently, thyme essential oils are not only effective acaricides reducing *Varroa* loads^20–22^, but also potential antimicrobials reducing bacteria loads of infected bees. However, repeated and permanent exposure should be avoided to prevent the rapid evolution of pathogen/parasite resistance, as shown recently for thymol and *C*. *bombi*^85^.

## Methods

### Phytochemical concentrations in nectar, pollen and leaves

Nectar and pollen samples of thyme were harvested in spring (May 2017) during flowering season. All thyme chemotypes originate from Southern France at CNRS (Montpellier, France) and were planted in 2013 (Halle (Saale), Germany). *T*. *vulgaris* plants (vegetative clones) of most chemotypes (C, T, G, A, and U) had enough nectar to produce 2–3 replicates per chemotype. Nectar samples were harvested with 1 µl micro-capillaries (Brand, Wertheim, Germany), and pooled among all individual plants of one chemotype. During harvesting, nectar was kept in glass vials on ice and then stored at −20 °C until measurement. Each pool consisted of 10 to 40 µl nectar. The L-chemotype provided only a very low amount of nectar which was sufficient for a single extraction and quantification. Pollen samples could only be harvested from the G-, A-, and L-chemotype as the C, T and U chemotypes only had female flowers which do not produce anthers with a pollen sac.

Pollen, more precisely anthers containing pollen producing microsporangia (from now on called pollen), was collected in glass vials by pinching the pollen sac with clean forceps together with as little amount of the filament as possible. Pollen grain sampling was not possible for the amounts needed to quantify terpenes. Each pollen sample consisted of a pool of one chemotype. The G-chemotype did not offer enough pollen to analyse multiple samples, it was however sufficient for a single extraction and quantification. During harvest, pollen was kept and stored under the same conditions as the nectar.

Nonyl acetate was used as internal standard and added to hexane before extraction (10 µg/ml, Sigma-Aldrich). Five to ten µl of each nectar sample were extracted in 20 µl hexane by vortexing for 2 min in glass micro tubes (100 µl). For extraction of pollen volatiles, 16–18 mg of pollen per sample were incubated in 200 µl hexane for 45 min at room temperature in glass vials with short interval mixing. For the nectar samples, extracts were frozen in liquid nitrogen and hexane removed from the solid phase. Pollen-extracts were centrifuged at 120 g for 1 min, followed by hexane removal from the pollen pellet. Nectar and pollen volatiles were identified with a gas chromatograph (GC-2010, Shimadzu) coupled to a mass spectrometer (GCMS-QP 2010 Plus, Shimadzu), using hydrogen as GC-MS carrier gas at a constant flow rate of 1 ml/min^86^. Quantification was performed by means of a flame ionization detector (FID-2010 Plus, Shimadzu). In both cases, 2 µl hexane extract were injected with an injector temperature of 220 °C in splitless mode. Separation and identification of all volatiles were performed as described earlier^86^, with the following GC-program: 40 °C for 3 min, first ramp 6 °C/min to 180 °C, second ramp 100 °C/min to 300 °C, final hold for 2 min. For GC-FID analyses, the same temperature program was used.

Young leaves from each of the six *T*. *vulgaris* chemotypes were ground to fine powder with mortar and pestle in liquid nitrogen. The powder (4–13 mg per sample) was mixed with 600 µl hexane (added internal standard: 10 µg/ml nonyl acetate) and incubated for 1 h at room temperature with short interval mixing. Afterwards, the extract was centrifuged at 120 g for 1 min, following hexane removal from the leaf pellet. GC-MS and GC-FID analysis of leaf volatiles were performed as described for nectar and pollen volatiles by using 1 µl hexane extract. Extraction was repeated for 3–4 plants of each chemotype as biological replicates.

In a final step, sensitivity of the GC-FID method was verified by determining method precision, substance recovery, the limit of detection (LOD) and limit of quantification (LOQ) for geraniol, exemplary for all nine thyme terpenes, even though we are aware that each substance might differ slightly for detector response. The same protocol as for substance determination from thyme nectar and 1 µl injection volume were used. In detail, three serial dilutions of geraniol (in hexane) were repeatedly quantified (1000, 750, 500, 250, 100, 50, 25, 12.5, 6.25, 3.125, 1.563, 0.781, 0.391, 0.195, 0.098, 0.049 ppm). Background noise (base line amplitude) was measured for the last 7 dilutions (with highest correlation of substance concertation and normalised peak height, *r* = 0.9998) to calculate LOD and LOQ.

Terpene quantification was implemented with high accuracy of method inter-day repeatability for substance detection (mean retention time SD of 0.2 sec) (Supplementary Table S3). Substance recovery for each dilution, in relation to the internal standard, was on average 83.7%, by excluding the last dilution (0.049) which was below the LOD (Supplementary Table S3). Nevertheless, high correlation between substance concentration and geraniol peak area was observed (n = 15, *r* = 0.9998). The limit of detection (0.059 ppm) and limit of quantification (0.072 ppm) for geraniol were an order of magnitude lower than the lowest value detected in all thyme samples (Table 1).

### Antibiotic potential of monoterpenes and their acetates

Bacteria species (all Gram-positive) were provided by the BCCM/LMG (Ghent University, Ghent, Belgium) and the Leibniz Institute DSMZ-German bacteria collection: *B*. *laterosporus* (DSM-25), *E*. *faecalis* (DSM-20478), *P*. *alvei* (DSM-29), *P*. *dendritiformis* (DSM-18844); Eva Forsgren (Swedish University of Agricultural Sciences, Uppsala, Sweden): *Bacillus pumilus* - strain SLU 119–12^7^; and Jean-Daniel Charrière (Swiss Bee Research Center, Agroscope, Bern, Switzerland): *Melissococcus plutonius* - strain 49.3^66^. They were cultivated at 35 °C or 30 °C (only *Pseudomonas fluorescens* (DSM-50090), see below) in standard medium as described earlier^7,30^. In the past, *Achromobacter eurydice* has also been mentioned as an EFB associated bacterium, as a so-called secondary invader^28^. However its role in the disease is still controversial^87^ and was not addressed in this study.

*Escherichia coli* (JM109, Promega) and *P*. *fluorescens* were included as non-pathogenic controls to compare the results for Gram-positive and Gram-negative (*E*. *coli*, *P*. *fluorescens*) bacteria.

The nine terpenes (thymol, carvacrol, geraniol, linalool, α-terpineol, trans-sabinene hydrate, geranyl acetate, α-terpinyl acetate and linalyl acetate) were all supplied from Sigma-Aldrich. All substances were solved or diluted with 100% DMSO (dimethylsulfoxide) (Sigma-Aldrich) to 600 mg/ml (60%) stock solutions, and diluted further (with 100% DMSO) to reach final concentrations of 0.02, 0.04, 0.2, 0.4, 2, 4, 20 and 40%. These solutions were stored at −20 °C to prevent evaporation. In the final growth inhibition assay, the latter solutions were diluted 40-times, which means that the final concentration per well (200 µl) was 5, 10, 50, 100, 500, 1000, 5000, 10000 and 15000 ppm. Final DMSO concentrations in the reaction volume used to estimate the antibacterial activity of each substance ranged from 1 to 2.5% (v/v). Consequently, controls with 1, 1.5, 2 and 2.5% DMSO were included as well. From previous studies it was known that the maximum non-inhibitory concentration of DMSO is 5% (v/v)^88,89^. pH shifts caused by the terpenes and their acetates may potentially inhibit the bacteria growth as well. To preclude this, the highest and lowest substrate and DMSO concentrations were tested using pH control strips (Macherey-Nagel, pH-Fix 2.0–9.0) and showed a pH of 7.0 to 7.5 for all tested substances and concentrations.

The bacteria growth inhibition assay (standard time-kill assay) was performed following the 96-well plate protocol of Erler and colleagues^7^, which monitors the complete bacterial growth curve and allows one to determine the starting point and linear slope of the logarithmic growth phase. Plates were incubated for 24 hours in a Synergy Mx microplate reader (BioTek, Winooski, VT, USA). *M*. *plutonius* was the only bacterium that needed to be cultivated in a CO_2_-incubator (10% CO_2_). Bacterial growth of this species was measured once a day (every 24 h) for 4 consecutive days using the same microplate reader, without shaking mode. All substances and controls (pure medium, medium with DMSO, medium with terpenes dissolved in DMSO) were tested with and without the respective bacterium. Substances and controls with bacterium were run in five replicates each, whereas negative controls (without bacterium) were run with two to three replicates each. Final growth curves were normalised to their respective negative controls. The slope of the log-phase was determined using a linear regression (with at least four data points and the highest correlation coefficient) and standard spreadsheet software. The bacterial growth inhibition was estimated with the formula described earlier^7^ and used to estimate the minimum inhibitory concentration (MIC), the relative half maximum inhibitory concentration (IC_50_, which is the same as the EC_50_ - half maximum effective concentration) and the maximum inhibitory concentration (IC_max_)^55^. Therefore, the growth inhibition values for the nine different concentrations (five per concentration, in %, Supplementary Data S1) were plotted against the terpene concentration (ppm as log-values), and fitted with at least 100 iterations to a non-linear (sigmoidal) dose-response curve with variable slope, as implemented in Origin^TM^ (Microcal^TM^ Origin^TM^, Version 5.0) (Fig. S1):

1 $$y=\frac{A1-A2}{\begin{array}{c}1+{e}^{(x-x0)/dx}\end{array}}+A2$$

with

$$\begin{array}{rcl}A1 & = & {\rm{lowest}}\,{\rm{growth}}\,{\rm{inhibition}}\,{\rm{after}}\,{\rm{fitting}}\\ A2 & = & {\rm{highest}}\,{\rm{growth}}\,{\rm{inhibition}}\,{\rm{after}}\,{\rm{fitting}}\,({\rm{usually}}\,100 \% )\\ x0 & = & {\rm{relative}}\,{{\rm{IC}}}_{50}({\rm{see}}\,{\rm{formula}}\,(2))\\ dx & = & {\rm{width}}\end{array}$$

All inhibitory concentrations (MIC, IC_50_, and IC_max_)^55^ were calculated using the linear part of the dose-response curve modelled by Origin^TM^ with at least 13 data points and the highest correlation coefficient. The MIC was generated with the *A*1 value and IC_max_ with the *A*2 value of the dose-response curve formula (1). For computing the relative IC_50_, the formula:

2 $$I{C}_{50}=(A1+A2)/2$$

was used.

The obtained y-values (*A*1, *A*2, IC_50_) were used in a converted linear function:

3 $$x=(y-b)/a$$

with

$$a={\rm{slope}}\,{\rm{of}}\,{\rm{the}}\,{\rm{linear}}\,{\rm{function}}$$

$$b={y}-{\rm{intercept}}\,{\rm{point}}$$

Finally, values were exponentiated (10^x^) to get inhibition values (MIC, IC_50_, IC_max_) in ppm. To visualize all results, IC_50_ values were log-transformed and a heat map was generated using MultiExperiment Viewer v. 4.9, according to the method described elsewhere^90^, with Manhattan distance and average linkage clustering to build dendrograms.

*M*. *plutonius* was the only strain where we could determine IC_50_ values only, but not MIC or IC_max_. Slope of the growth curves were determined using a linear regression (between 2 days, one without and one with detectable bacteria, usually between days 2 and 3) and standard spreadsheet software. All following steps were the same as described above. Due to high variance among replicates, the median among replicates was used to estimate IC_50_ values.

### Antennal response

Electroantennogram (EAG) recording was conducted according to the BEEBOOK recommendations^91^. Newly emerged worker bees were taken directly from the incubator (1 day old)^8^, whereas forager honey bees were caught at the flight entrance, on their way back to the hive. All measurements were conducted at the end of summer, outside the main flowering season of thyme to prevent predisposition of the foragers.

Antennal response (in mV) was recorded for seven bees per group (newly emerged, forager), with a Syntech IDAC-232 system and automatic base line control. Test odorants were applied on single antennae (entire antennae with cut tip - two segments, and cut as close as possible to the bees’ head) inserted into two glass capillaries filled with bee-Ringer^92^. The bees’ antennae were tested for background EAG response using two background stimuli: (1) air stimulus and (2) air stimulus that contained DMSO. For testing odorant response, odorants were delivered successively 5–7-times as discrete stimuli with 0.5 s in duration and minimum 3–10 s intervals between stimulations. The order of presentation of the different substances was randomised with DMSO background controls after half of the test odorants were measured and at the end of each antennae assay. Throughout all measurements, constant humidified air flow was guaranteed with 7 ml/s. Antennae response data were analysed using EAGPro V2.0 (Syntech, Hilversum) software.

Geometric means of each substance (across the 5–7 technical replicates) were normalised to their respective DMSO controls (geometric means), measured after four substances tested per antennae. The response value data set was first tested for extreme outliers (box-plots). Extreme values were defined as data points which are outside the three box length range from the upper and lower value of the box (25–75% percentile range). This led to the complete exclusion of bee number 6 in the newly emerged bee data set, and 2 values for linalyl acetate and 1 value for trans-sabinene hydrate in the forager bee data set. After correction for extremes, data sets of forager and newly emerged bees (Supplementary Data S1) were combined to estimate the general impact of ‘age’ (newly emerged workers *vs*. forager), ‘substance’ and the interaction of both, using a general linear model with Bonferroni post-hoc tests. In a second step, antennal response between substances, but within newly emerged worker or forager bees, was compared using Kruskal-Wallis ANOVAs, with post-hoc multiple comparisons of mean ranks of all pairs of groups, to verify grouping of response patterns related to chemical structure (e.g. acetates *vs*. non-acetates, phenolic *vs*. non-phenolic). All statistical tests were conducted using STATISTICA 8.0 (StatSoft; Tulsa, OK, USA).

### Ethics statement

Endangered or protected species were not used in this study. Experiments and observations conform with the laws of Germany in relation to animal protection. No specific ethics certification is required for this research.

## Electronic supplementary material

Supplementary Information and Material

Dataset 1
