# Supplementary material for: The terpenes of leaves, pollen, and nectar of thyme (Thymus vulgaris) inhibit growth of bee disease-associated microbes
Source: Sci Rep. 2018 Oct 2;8:14634. doi: 10.1038/s41598-018-32849-6 (PMC6168512; doi:10.1038/s41598-018-32849-6)
Supplement: Supplementary file 1 — Supplementary Information and Material [file 41598_2018_32849_MOESM1_ESM.docx]

**Supplementary Information and Material**

**The terpenes of leaves, pollen, and nectar of thyme (*Thymus vulgaris*) inhibit growth of bee disease-associated microbes**

Natalie Wiese, Juliane Fischer, Jenifer Heidler, Oleg Lewkowski, Jörg Degenhardt, Silvio Erler

**Figure S1**: Sigmoidal dose-response curve according to du Toit and Rautenbach (2000), estimated for *B. laterosporus* and thymol. Bacterial growth inhibition was calculated for the minimum inhibitory concentration (MIC), the relative half maximum inhibitory concentration (IC_50_) and the maximum inhibitory concentration (IC_max_) using the formula given in Material and Methods.


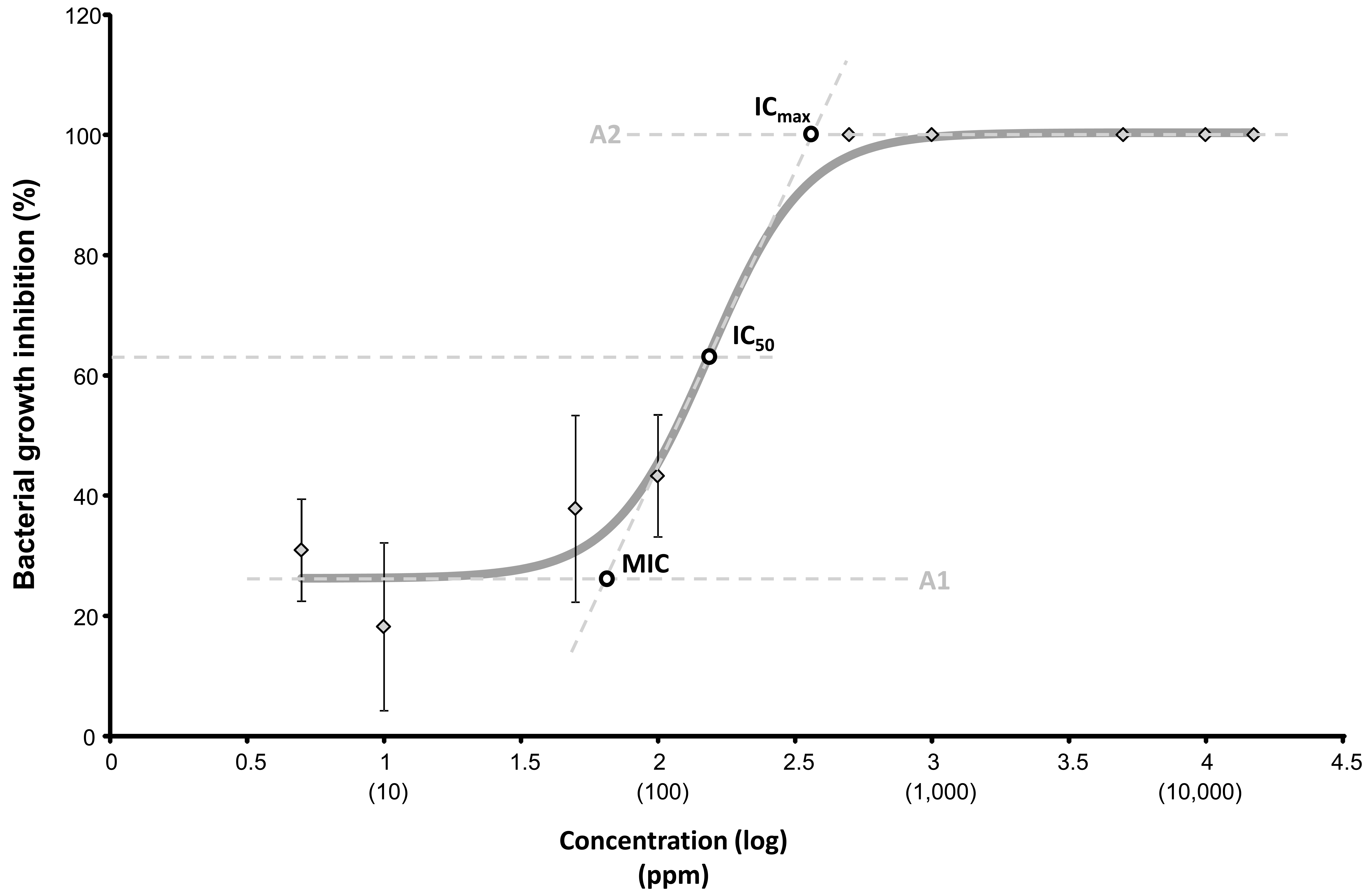


**Reference**

du Toit EA, Rautenbach M (2000) A sensitive standardised micro-gel well diffusion assay for the determination of antimicrobial activity. *J Microbiol Methods* **42**: 159-165

**Figure S2**: Chemical structures and relatedness of the compounds. Structures are originally from PubChem Open Chemistry Database (National Center for Biotechnology Information, U.S. National Library of Medicine) (Kim *et al*. 2016).





**Reference**

Kim, S., Thiessen, P.A., Bolton, E.E., Chen, J., Fu, G., Gindulyte, A., Han, L., He, J., He, S., Shoemaker, B.A., Wang, J., Yu, B., Zhang, J., Bryant, S.H. (2016) PubChem Substance and Compound databases. *Nucleic Acids Res*, **44**: D1202-13. doi: 10.1093/nar/gkv951

**Table S1:** Summary of *T. vulgaris* terpene antimicrobial activity (in ppm) towards several Gram-positive (*M. plutonius* and bacteria associated with European foulbrood disease) and Gram-negative bacteria, expressed in MIC (minimum inhibitory concentration), IC_50_ (relative half maximum inhibitory concentration) and IC_max_ (maximum inhibitory concentration). Values are given as means ± standard error.

| **Substance** |  | ***E. coli*** | ***P. fluorescens*** | ***B. pumilus*** | ***E. faecalis*** | ***P. alvei*** | ***P. dendritiformis*** | ***B. laterosporus*** | ***M. plutonius*^Δ^** |
| --- | --- | --- | --- | --- | --- | --- | --- | --- | --- |
| **Carvacrol** | MIC | 135.5 ± 1.03 | 285.9 ± 0.71 | 804.2 ± 0.47 | 504.8 ± 0.33 | 101.6 ± 0.14 | 307.6 ± 0.45 | 375.5 ± 0.54 | n.d. |
|  | IC_50_ | 184.7 ± 2.61 | 301.4 ± 0.75 | 819.9 ± 0.55 | 536.4 ± 0.38 | 105.9 ± 0.12 | 321.6 ± 0.47 | 385.9 ± 0.56 | 453.5 |
|  | IC_max_ | 255.4 ± 1.74 | 317.7 ± 0.79 | 835.9 ± 0.63 | 569.9 ± 0.43 | 110.3 ± 0.12 | 336.2 ± 0.49 | 396.7 ± 0.57 | n.d. |
| **Thymol** | MIC | 103.3 ± 0.75 | 349.2 ± 0.81 | 709.6 ± 0.51 | 856.1 ± 0.9 | 135.1 ± 1.84 | 102.8 ± 0.14 | 61.7 ± 4.3 | n.d. |
|  | IC_50_ | 163.6 ± 1.09 | 360.8 ± 0.84 | 717.2 ± 0.58 | 869.3 ± 1.05 | 178.8 ± 2.83 | 106.9 ± 0.13 | 158.2 ± 9.8 | 102.1 |
|  | IC_max_ | 259.1 ± 1.57 | 372.8 ± 0.86 | 724.8 ± 0.65 | 882.7 ± 1.2 | 236.6 ± 2.28 | 111.1 ± 0.12 | 405.2 ± 22.0 | n.d. |
| **alpha-Terpineol** | MIC | 487.8 ± 1.01 | 506.1 ± 1.65 | 616.9 ± 37.77 | n.e. | 294.2 ± 0.6 | 489.5 ± 1.06 | 468.6 ± 4.67 | n.d. |
|  | IC_50_ | 520.7 ± 1.16 | 539.7 ± 1.9 | 1077.1 ± 368.39 | n.e. | 308.2 ± 0.6 | 521.1 ± 1.22 | 509.8 ± 5.48 | 81.7 |
|  | IC_max_ | 555.8 ± 1.33 | 575.6 ± 2.17 | 3161.3 ± 30.33 | n.e. | 322.9 ± 0.6 | 554.6 ± 1.38 | 554.6 ± 6.39 | n.d. |
| **Linalool** | MIC | 514.6 ± 0.47 | 973.5 ± 1.81 | 5129.1 ± 7.38 | n.e. | 521.1 ± 0.9 | 479.15^x^ ± 57.18 | 358.7 ± 0.78 | n.d. |
|  | IC_50_ | 544.6 ± 1.93 | 1010.6 ± 2.15 | 5435.8 ± 13.42 | n.e. | 552.3 ± 1.03 | 3135.23^x^ ± 436.55 | 370.3 ± 0.81 | 1627.3 |
|  | IC_max_ | 579.4 ± 0.61 | 1049.1 ± 2.52 | 5760.7 ± 20.17 | n.e. | 585.4 ± 1.17 | 20514.62^x^ ± 3272.05 | 382.3 ± 0.84 | n.d. |
| **Geraniol** | MIC | 529.8 ± 0.65 | 5155.7 ± 6.03 | 7875.6 ± 6.03 | 5193.6 ± 5.18 | 117.3 ± 2.5 | 100.9 ± 0.16 | 100.9 ± 0.16 | n.d. |
|  | IC_50_ | 559.7 ± 0.74 | 5483.9 ± 10.86 | 8040.9 ± 10.86 | 5512.9 ± 9.49 | 180.1 ± 3.56 | 106.1 ± 0.14 | 105.9 ± 0.15 | 433.5 |
|  | IC_max_ | 591.2 ± 0.84 | 5833.1 ± 15.91 | 8209.6 ± 15.91 | 5851.9 ± 14.31 | 276.5 ± 5.05 | 111.5 ± 0.13 | 111.01 ± 0.14 | n.d. |
| **trans-Sabinene hydrat** | MIC | 4790.3* ± 10.53 | 927.3* ± 1.21 | >5000 | >5000 | 99.1 ± 0.19 | 71.1 ± 7.54 | 465.8 ± 1.31 | n.d. |
|  | IC_50_ | 5044.4* ± 29.22 | 961.8* ± 1.43 | >5000 | >5000 | 102.9 ± 0.16 | 400.3 ± 41.82 | 498.7 ± 1.51 | 139.1 |
|  | IC_max_ | 5428.9* ± 74.57 | 997.6* ± 1.68 | >5000 | >5000 | 106.8 ± 0.16 | 2253.8 ± 231.67 | 534.0 ± 1.73 | n.d. |
| **Geranyl acetate** | MIC | n.e. | n.e. | n.e. | n.e. | n.e. | n.e. | n.e. | n.d. |
|  | IC_50_ | n.e. | n.e. | n.e. | n.e. | n.e. | n.e. | n.e. | 2951.6 |
|  | IC_max_ | n.e. | n.e. | n.e. | n.e. | n.e. | n.e. | n.e. | n.d. |
| **Linalyl acetate** | MIC | n.e. | n.e. | n.e. | n.e. | n.e. | n.e. | n.e. | n.d. |
|  | IC_50_ | n.e. | n.e. | n.e. | n.e. | n.e. | n.e. | n.e. | 8617.9* |
|  | IC_max_ | n.e. | n.e. | n.e. | n.e. | n.e. | n.e. | n.e. | n.d. |
| **Terpinyl acetate** | MIC | n.e. | n.e. | n.e. | n.e. | n.e. | n.e. | n.e. | n.d. |
|  | IC_50_ | n.e. | n.e. | n.e. | n.e. | n.e. | n.e. | n.e. | 6992.8 |
|  | IC_max_ | n.e. | n.e. | n.e. | n.e. | n.e. | n.e. | n.e. | n.d. |

n.e.: no growth inhibitory effect detectable; n.d.: not determined; ^Δ^: standard errors not determined; ^*^: Extrapolated data were used for cases where the following higher concentration was not evaluable (trans-sabinene hydrate only) or not evaluated (linalool only, complete inhibition expected with 50000 ppm). This means we expected complete inhibition, when the last measurable concentration inhibited by at least 30%.; ^x^: All three values are slightly overestimated, in particular the IC_max_, as extrapolated data needed to be used to calculate all of them in Origin. The real IC_max_ should be somewhere between 10000 and 15000 ppm.; >5000: growth inhibition was not measurable at higher concentrations (insolubility of the substance) and no complete growth inhibition was detected using 5000 ppm

**Table 2:** Delay of bacterial growth (start of log phase) for all terpene concentrations given in hours ± SD. All data points are normalized to their respective positive controls (medium and bacteria without substrate).

| **Substrate** | **Concentration (ppm)** | ***E. coli*** | ***P. fluorescens*** | ***B. pumilus*** | ***E. faecalis*** | ***P. alvei*** | ***P. dendritiformis*** | ***B. laterosporus*** |
| --- | --- | --- | --- | --- | --- | --- | --- | --- |
| **Carvacrol** | 15,000 | † | † | † | † | † | † | † |
|  | 10,000 | † | † | † | † | † | † | † |
|  | 5,000 | † | † | † | † | † | † | † |
|  | 1,000 | † | † | † | † | † | † | † |
|  | 500 | † | † | 17.05 ± 0.54 | 11.71 ± 0.45 | † | † | † |
|  | 100 | 0.11 ± 0.11 | -1.2 ± 0.59 | 0.45 ± 0.11 | 0.06 ± 0 | 5.9 ± 0.29 | 0.26 ± 0.54 | 3.1 ± 0.38 |
|  | 50 | -0.29 ± 0.14 | -1 ± 0.64 | -0.1 ± 0.14 | 0.06 ± 0 | 2.65 ± 0.14 | 0.11 ± 0.33 | 0.55 ± 0.11 |
|  | 10 | -0.04 ± 0.14 | -1.7 ± 0.44 | -0.05 ± 0.11 | 0.06 ± 0 | 0.6 ± 0.14 | 0 ± 0.49 | 0 ± 0 |
|  | 5 | -0.04 ± 0.14 | -1.75 ± 0.85 | -0.15 ± 0.14 | 0.01 ± 0.11 | 0.5 ± 0.18 | 0.21 ± 0.22 | 0 ± 0 |
|  | control (1) | 0.44 ± 0.14 | 0 ± 0 | 0.06 ± 0 | 0.25 ± 0.14 | -0.25 ± 0.13 | 0.31 ± 0.38 | 0 ± 0 |
|  | control (1.5) | -0.5 ± 0.24 | -0.06 ± 0.66 | 0.06 ± 0 | 0.19 ± 0.13 | 0.06 ± 0.13 | 0.19 ± 0.38 | 0 ± 0.2 |
|  | control (2) | -0.06 ± 0 | 0.38 ± 0.48 | 0.31 ± 0 | 0.38 ± 0 | 0 ± 0 | 0.38 ± 0.25 | 0.13 ± 0.14 |
|  | control (2.5) | -0.38 ± 0.13 | 0.75 ± 0 | 0.56 ± 0 | 0.31 ± 0,13 | 0.25 ± 0 | 0.19 ± 0.43 | 0.25 ± 0 |
| **Thymol** | 15,000 | † | † | † | † | † | † | † |
|  | 10,000 | † | † | † | † | † | † | † |
|  | 5,000 | † | † | † | † | † | † | † |
|  | 1,000 | † | † | † | † | † | † | † |
|  | 500 | † | † | 15.5 ± 1.51 | 10.05 ± 0.78 | † | † | † |
|  | 100 | 0.19 ± 0 | 0.85 ± 0.22 | 1.45 ± 0.11 | 0.35 ± 0.14 | 8.04 ± 0.29 | 1.13 ± 0.25 | 0 ± 0.74 |
|  | 50 | -0.01 ± 0.11 | 0.75 ± 0 | 0.4 ± 0.14 | 0.1 ± 0.14 | 3.44 ± 0.31 | 0.08 ± 0.33 | 0 ± 0.34 |
|  | 10 | 0.09 ± 0.14 | 0.45 ± 0.21 | 0.05 ± 0.14 | 0.15 ± 0.14 | 1.09 ± 0.14 | 0 ± 0.22 | 0 ± 0.18 |
|  | 5 | 0.09 ± 0.14 | 0.65 ± 0.14 | 0.15 ± 0.14 | 0.25 ± 0 | 0.74 ± 0.32 | 0 ± 0.38 | 0 ± 0.54 |
|  | control (1) | -0.19 ± 0 | -0.19 ± 0.31 | 0.19 ± 0.24 | 0.13 ± 0 | 0.38 ± 0.13 | 0 ± 0.14 | 0 ± 0.69 |
|  | control (1.5) | -0.19 ± 0 | -0.25 ± 0.35 | 0.31 ± 0.13 | 0.19 ± 0.13 | 0.44 ± 0.21 | 0.06 ± 0.24 | 1.25 ± 0.83 |
|  | control (2) | -0.06 ± 0.14 | 0.88 ± 0.14 | -0.19 ± 0.31 | 0.19 ± 0.13 | 0.31 ± 0.14 | 0.31 ± 0.24 | 0.25 ± 0.85 |
|  | control (2.5) | -0.13 ± 0.13 | 0.75 ± 0 | 0.5 ± 0.5 | 0.13 ± 0 | 0.25 ± 0.24 | 0.25 ± 0.14 | 1.38 ± 0.24 |
| **alpha-Terpineol** | 15,000 | † | † | † | 13.68 ± 2.43 | † | † | † |
|  | 10,000 | † | † | † | 5.63 ± 1.66 | † | † | † |
|  | 5,000 | † | † | † | 3.25 ± 0.5 | † | † | † |
|  | 1,000 | † | † | 13.28 ± 1.04 | 0.75 ± 0 | † | † | † |
|  | 500 | 0.5 ± 0 | 1.31 ± 0.35 | 1.08 ± 0.11 | 0.4 ± 0.14 | † | 1.6 ± 0.76 | 4.26 ± 0.48 |
|  | 100 | 0.1 ± 0.14 | 0.56 ± 0.18 | 1.33 ± 0.11 | 0.25 ± 0 | 5.51 ± 0.11 | 0.45 ± 0.11 | 0 ± 1.27 |
|  | 50 | 0.05 ± 0.11 | 0.66 ± 0.14 | 1.08 ± 0.11 | 0.25 ± 0 | 3.31 ± 0 | 0.05 ± 0.27 | 0 ± 0.65 |
|  | 10 | 0.05 ± 0.11 | 0.56 ± 0.25 | 1.03 ± 0.14 | 0.15 ± 0.14 | 1.56 ± 0 | 0 ± 0.14 | 0 ± 0.98 |
|  | 5 | 0.05 **±** 0.11 | 0.76 ± 0.44 | 0.98 ± 0.14 | 0 ± 0 | 1.06 ± 0 | 0 ± 0 | 0 ± 0.57 |
|  | control (1) | -0.06 ± 0.13 | 0.19 ± 1.11 | -0.19 ± 1.01 | 0.13 ± 0.14 | -0.19 ± 0.13 | 0.06 ± 0.14 | 0.94 ± 0.29 |
|  | control (1.5) | 0 ± 0 | 0.88 ± 0.35 | 1.13 ± 0.24 | 0.13 ± 0.14 | 0.38 ± 0 | 0.13 ± 0.13 | 0 ± 1.45 |
|  | control (2) | 0 ± 0 | 2 ± 0.14 | 1.44 ± 0 | 0.25 ± 0 | 0.06 ± 0.13 | 0.19 ± 0 | 1.5 ± 0.43 |
|  | control (2.5) | 0 ± 0 | 1.81 ± 0.24 | 1.56 ± 0.14 | 0.25 ± 0 | 0.56 ± 0.13 | 0.19 ± 0.20 | 1.38 ± 0.77 |
| **Linalool** | 15,000 | † | † | † | 5.64 ± 2.73 | † | † | † |
|  | 10,000 | † | † | † | 2.81 ± 0.87 | † | 19.25 ± 1.48 | † |
|  | 5,000 | † | † | 11.25 ± 0.95 | 0.55 ± 0.11 | † | 15.3 ± 0.48 | † |
|  | 1,000 | † | 6.51 ± 0.69 | 7.66 ± 0.72 | 0.25 ± 0 | † | 12.83 ± 0.27 | † |
|  | 500 | 0 ± 0 | 0.71 ± 0.39 | 0.56 ± 0 | 0 ± 0 | 7.7 ± 0.21 | 1.23 ± 0.14 | † |
|  | 100 | 0.05 ± 0.11 | 0.21 ± 0.14 | 0.06 ± 0 | 0 ± 0 | 2.3 ± 0.11 | 0.23 ± 0.14 | 1.1 ± 0.68 |
|  | 50 | -0.05 ± 0.11 | 0.51 ± 0.21 | 0.01 ± 0.11 | 0 ± 0 | 1.55 ± 0.33 | 0.08 ± 0.11 | 1.25 ± 0.47 |
|  | 10 | 0 ± 0 | 0.21 ± 0.14 | 0.16 ± 0.14 | 0.05 ± 0.11 | 0.65 ± 0.14 | 0.08 ± 0.21 | 0.2 ± 0.11 |
|  | 5 | 0 ± 0 | 0.81 ± 0 | 0.06 ± 0 | 0 ± 0 | 0.85 ± 0.14 | 0 ± 0.23 | 0.55 ± 0.11 |
|  | control (1) | -0.56 ± 0.13 | 1.56 ± 0.38 | -0.56 ± 0.35 | 0.06 ± 0.13 | 0 ± 0 | 0 ± 0.14 | 0.13 ± 0 |
|  | control (1.5) | -0.44 ± 0.13 | 1.81 ± 0.13 | -0.19 ± 0.14 | 0.19 ± 0.13 | 0.13 ± 0.14 | 0 ± 0.14 | 0.19 ± 0.13 |
|  | control (2) | -0.19 ± 0.13 | 2.25 ± 0 | -0.06 ± 0 | 0.25 ± 0 | 0 ± 0 | 0.25 ± 0.20 | 0.63 ± 0.29 |
|  | control (2.5) | -0.25 ± 0 | 2.69 ± 0,13 | 0.13 ± 0.13 | 0.25 ± 0 | 0 ± 0 | 0.13 ± 0.14 | 0.5 ± 0.14 |
| **Geraniol** | 15,000 | † | † | † | † | † | † | † |
|  | 10,000 | † | † | † | † | † | † | † |
|  | 5,000 | † | 12.75 ± 1.33 | 13.38 ± 0.61 | 9.8 ± 0.59 | † | † | † |
|  | 1,000 | † | 7.96 ± 0.74 | 7.5 ± 0.31 | 4.4 ± 0.33 | † | † | † |
|  | 500 | 7.95 ± 0.65 | 6.26 ± 0.21 | 7.75 ± 0.21 | 1.85 ± 0.14 | † | † | † |
|  | 100 | 0.2 ± 0.11 | -0.24 ± 0.21 | -0.15 ± 0.14 | 0.15 ± 0.14 | 1.5 ± 0 | 0 ± 0 | 0.6 ± 0.14 |
|  | 50 | 0.05 ± 0.11 | -0.74 ± 0.27 | -0.4 ± 0.22 | 0 ± 0.35 | 0.8 ± 0.11 | 0 ± 0.33 | 0 ± 0.11 |
|  | 10 | 0.25 ± 0 | -0.14 ± 0.11 | -0.4 ± 0.14 | 0.05 ± 0.11 | 0.2 ± 0.11 | 0.11 ± 0.11 | 0.25 ± 0 |
|  | 5 | 0.2 ± 0.11 | 0.01 ± 0.27 | -0.45 ± 0.27 | 0.05 ± 0.11 | 0.25 ± 0 | 0 ± 0.22 | 0 ± 0.18 |
|  | control (1) | -0.06 ± 0.14 | 0.56 ± 0.14 | -0.06 ± 0.43 | 0.13 ± 0.13 | 0.13 ± 0.14 | 0 ± 0.2 | 0 ± 0 |
|  | control (1.5) | -0.06 ± 0.14 | 0.81 ± 0.14 | 0.63 ± 0.13 | 0.19 ± 0 | 0.13 ± 0.43 | 0.06 ± 0.14 | 0.19 ± 0 |
|  | control (2) | 0.13 ± 0.13 | 1.44 ± 0.29 | 0.94 ± 0.14 | 0.19 ± 0 | 0.38 ± 0.14 | 0 ± 0.31 | 0.19 ± 0 |
|  | control (2.5) | 0.06 ± 0 | 1.86 ± 0.24 | 1.06 ± 0 | 0.19 ± 0 | 0.25 ± 0 | 0.13 ± 0.13 | 0.44 ± 0 |
| **trans-Sabinene hydrate** | 15,000 | **n.m.** | **n.m.** | **n.m.** | **n.m.** | † | † | † |
|  | 10,000 | **n.m.** | **n.m.** | **n.m.** | **n.m.** | † | † | † |
|  | 5,000 | 5.3 ± 0.33 | **n.m.** | 12.75 ± 0.47 | 0.76 ± 0.11 | † | † | † |
|  | 1,000 | 0.05 ± 0.11 | 12.38 ± 0.51 | 4.91 ± 0.29 | 0.75 ± 0 | † | 15.8 ± 0.56 | † |
|  | 500 | 0 ± 0 | 1.26 ± 0.11 | 1.01 ± 0.11 | 0.3 ± 0.11 | † | 4.2 ± 0.27 | 1.29 ± 1.1 |
|  | 100 | 0 ± 0 | 0.71 ± 0.29 | 0.21 ± 0.14 | 0.25 ± 0 | 1.91 ± 0.14 | 0 ± 0.22 | 0 ± 1.16 |
|  | 50 | 0 ± 0 | 1.11 ± 0.21 | 0.06 ± 0 | 0.25 ± 0 | 0.81 ± 0.18 | 0 ± 0.29 | 0.19 ± 0.71 |
|  | 10 | 0 ± 0 | 0.96 **±** 0.22 | 0.01 ± 0.11 | 0.25 ± 0 | 0.11 ± 0.21 | 0 ± 0.29 | 0 ± 0.62 |
|  | 5 | 0 ± 0 | 0.81 ± 0.18 | 0.06 ± 0 | 0.2 ± 0.11 | 0.01 ± 0.11 | 0 ± 0.11 | 0 ± 0.27 |
|  | control (1) | -0.13 ± 0.14 | 1 ± 0.43 | 0.38 ± 0 | 0 ± 0 | 0.38 ± 0.13 | 0 ± 0.14 | 0 ± 0.13 |
|  | control (1.5) | 0 ± 0 | 0.88 ± 0.35 | 0.38 ± 0 | 0.06 ± 0.13 | 0.06 ± 0.25 | 0 ± 0.51 | 0 ± 0.43 |
|  | control (2) | 0 ± 0 | 1.75 ± 0.25 | 0.38 ± 0 | -0.06 ± 0.13 | 0.13 ± 0.13 | 0 ± 0.2 | 0.31 ± 0.43 |
|  | control (2.5) | 0 ± 0 | 1.56 ± 0.24 | 0.56 ± 0.13 | 0 ± 0 | 0.38 ± 0.13 | 0.13 ± 0.2 | 0.5 ± 0.52 |
| **Geranyl acetate** | 15,000 | 0.43 ± 0.21 | 1.74 ± 0.11 | 1.75 ± 0.18 | 0.45 ± 0.67 | 8.6 ± 2.4 | 5.49 ± 0.45 | 2.3 ± 0.48 |
|  | 10,000 | 0.25 ± 0.18 | 0.58 ± 0.21 | 1.96 ± 0.14 | 0.63 ± 0.39 | 4.8 ± 1.33 | 8.7 ± 0.48 | 0.34 ± 0.52 |
|  | 5,000 | 0.2 ± 0.11 | 0.03 ± 0.14 | 1.4 ± 0.22 | 0.65 ± 0.23 | 9.95 ± 1.37 | 9.63 ± 0.47 | 1.99 ± 0.45 |
|  | 1,000 | 0 ± 0 | 0.26 ± 0.11 | 0.7 ± 0.11 | 0.5 ± 0 | 6.63 ± 0.27 | 7.38 ± 0.18 | 0.65 ± 0.42 |
|  | 500 | 0.1 ± 0.14 | 0.06 ± 0 | 0.7 ± 0.11 | 0.5 ± 0 | 6.44 ± 0 | 6.63 ± 0.53 | 0.7 ± 0.41 |
|  | 100 | 0 ± 0 | 0.26 ± 0.11 | 0.2 ± 0.11 | 0.4 ± 0.14 | 2.18 ± 0 | 4.68 ± 0.11 | 0.5 ± 0.4 |
|  | 50 | 0 ± 0 | 0.06 ± 0.31 | -0.3 ± 0.11 | 0.3 ± 0.11 | 0 ± 0.43 | 1.89 ± 0.18 | 0.25 ± 0.31 |
|  | 10 | 0 ± 0 | 0.11 ± 0.27 | 0 ± 0 | 0.25 ± 0 | 0 ± 0 | 0.03 ± 0.22 | 0 ± 0.22 |
|  | 5 | 0.25 ± 0 | 0.21 ± 0.14 | -0.05 ± 0.11 | 0.25 **±** 0 | 0 ± 0.18 | 0.28 ± 0.29 | 0 ± 0.14 |
|  | control (1) | -0.31 ± 0.14 | 0.13 ± 0.66 | 0.25 ± 0 | 0 ± 0 | 0 ± 0 | 0.19 ± 0.24 | 0 ± 0.2 |
|  | control (1.5) | -0.19 ± 0 | 1.44 ± 0.48 | 0.44 ± 0.13 | -0.13 ± 0.14 | 0.06 ± 0.21 | 0.13 ± 0.35 | 0 ± 1.48 |
|  | control (2) | -0.19 ± 0 | 2.19 ± 0.14 | 0.5 ± 0 | 0 ± 0 | 0 ± 0 | 0.5 ± 0.25 | 0 ± 0.13 |
|  | control (2.5) | -0.19 ± 0 | 2.25 ± 0.24 | 0.75 ± 0 | 0 ± 0 | 0.06 ± 0.11 | 0.25 ± 0.25 | 0 ± 1.02 |
| **Linalyl acetat** | 15,000 | 0 ± 0 | 0.3 ± 0.11 | 1.3 ± 0.11 | 0.34 ± 0.22 | 5.91 ± 0.58 | 2.83 ± 0.51 | 0.39 ± 0.48 |
|  | 10,000 | 0.2 ± 0.11 | -0.16 ± 0.48 | 1.1 ± 0.14 | 0.4 ± 0.14 | 5.76 ± 0.41 | 2.05 ± 0.48 | 0 ± 0.21 |
|  | 5,000 | -0.03 ± 0.14 | -0.06 ± 0 | 0.89 ± 0.21 | 0.48 ± 0.34 | 8.45 ± 0.48 | 1.65 ± 0.14 | 0 ± 0.14 |
|  | 1,000 | 0.06 ± 0.18 | 0.06 ± 0.18 | 0.28 ± 0.14 | 0.68 ± 0.11 | 6.71 ± 0.42 | 0.88 ± 0.18 | 0 ± 0.45 |
|  | 500 | 0.11 ± 0.11 | -0.09 ± 0.38 | 0.38 ± 0 | 0.73 ± 0.14 | 7.27 ± 0.21 | 0.93 ± 0.11 | 0 ± 0.14 |
|  | 100 | 0.16 ± 0.14 | 0.06 ± 0 | -0.13 ± 0.18 | 0.53 ± 0.14 | 3.91 ± 0.14 | 1.18 ± 0.11 | 0 ± 0.51 |
|  | 50 | 0.06 ± 0 | 0.31 ± 0 | -0.13 ± 0.18 | 0.63 ± 0 | 0.61 ± 0.11 | 0.48 ± 0.22 | 0 ± 0.45 |
|  | 10 | 0.06 ± 0 | 0.16 ± 0.22 | -0.28 ± 0.14 | 0.63 ± 0 | 0.01 ± 0.11 | 0 ± 0.33 | 0 ± 0.21 |
|  | 5 | 0.06 ± 0 | 0.26 ± 0.11 | -0.23 0.14 | 0.48 ± 0.14 | 0 ± 0.14 | 0 ± 0.11 | 0 ± 0.52 |
|  | control (1) | -0.19 ± 0 | 1.19 ± 0.21 | 0.06 ± 0 | 0.13 ± 0.24 | 0 ± 0.13 | 0 ± 0.59 | 0.63 ± 0.13 |
|  | control (1.5) | -0.19 ± 0 | 1.5 ± 0.31 | 0.31 ± 0.21 | 0.06 ± 0 | 0.13 ± 0.13 | 0 ± 0.20 | 0.88 ± 0.13 |
|  | control (2) | -0.06 ± 0.14 | 2 ± 0.13 | 0.38 ± 0.13 | -0.06 ± 0.14 | 0 ± 0 | 0 ± 0.20 | 0.5 ± 0.52 |
|  | control (2.5) | -0.25 ± 0.13 | 2.13 ± 0.24 | 0.69 ± 0.14 | -0.06 ± 0.25 | 0.13 ± 0.24 | 0.25 ± 0.14 | 4.56 ± 1 |
| **Terpinyl acetate** | 15,000 | 0.23 ± 0.14 | 1.03 ± 0.22 | 3.18 0.94 | 0.66 ± 0.14 | 13.4 ± 2.26 | 11.48 ± 3.9 | 1.59 ± 0.52 |
|  | 10,000 | 0.38 ± 0 | 1.13 ± 0.18 | 2.75 0.47 | 0.2 ± 0.11 | 10.2 ± 1.94 | 10.34 ± 1.98 | 4.27 ± 0.11 |
|  | 5,000 | -0.06 ± 0 | 0.31 ± 0 | 3.05 0.11 | 0.53 ± 0.14 | 20.83 ± 1 | 7.91 ± 0.58 | 1.05 ± 0.48 |
|  | 1,000 | 0.1 ± 0.14 | 0.13 ± 0.18 | 2.04 0.14 | 0.5 ± 0 | 19,95 ± 1.21 | 5.63 ± 0.35 | 1.71 ± 0.34 |
|  | 500 | 0.05 ± 0.11 | 0.58 ± 0.27 | 1.99 0.11 | 0.45 ± 0.11 | 16.7 ± 2 | 5.63 ± 0.59 | 1.31 ± 0.27 |
|  | 100 | 0 ± 0 | 0.43 ± 0.21 | 0.44 ± 0 | -0.05 ± 0.11 | 4.65 ± 0.14 | 4.23 ± 0.22 | 1.16 ± 0.45 |
|  | 50 | 0 ± 0 | 0.63 ± 0.18 | -0.19 ± 0.14 | -0.25 ± 0.18 | 0.15 ± 0.14 | 1.28 ± 0.23 | 0 ± 0 |
|  | 10 | 0.05 ± 0.11 | 0.48 ± 0.22 | -0.17 ± 0.14 | -0.35 ± 0.14 | 0 ± 0.11 | 0.23 ± 0.14 | 0 ± 0 |
|  | 5 | 0 ± 0 | 0.58 ± 0.11 | -0.06 ± 0 | -0.45 ± 0.11 | 0 ± 0.11 | 0.03 ± 0.14 | 0 ± 0.21 |
|  | control (1) | 0.5 ± 0.14 | 1.06 ± 0.43 | 0.13 ± 0.14 | 0.13 ± 0.13 | 0 ± 0 | 0.25 ± 0.14 | 0.1 ± 0.24 |
|  | control (1.5) | 0.25 ± 0.32 | 1.06 ± 0.25 | 0.25 ± 0 | 0.19 ± 0 | 0 ± 0 | 0.19 ± 0.13 | 0.19 ± 0.13 |
|  | control (2) | 0.69 ± 0.13 | 2.13 ± 0.13 | 0.25 ± 0 | 0.31 ± 0.14 | 0.25 ± 0 | 0.31 ± 0.31 | 0.5 ± 0.29 |
|  | control (2.5) | 0.63 ± 0 | 2.31 ± 0.25 | 0.56 ± 0.13 | 0.44 ± 0 | 0.25 ± 0 | 0.25 ± 0.43 | 0.94 ± 0.13 |

control (1-2.5): bacterial growth control, only with DMSO in final concentrations of 1-2.5%; † : no bacterial growth detectable within 24 h; n.m.: bacterial growth was not measurable due to insolubility of the substance

**Table S3:** Validation parameters for geraniol quantification using GC-FID.

| **Concentration (ppm)** | **Retention time (min)** | | **Recovery (%)** |
| --- | --- | --- | --- |
|  | **Average** | **SD** |  |
| 1000 | 18.57 | 0.012 | 0.90 |
| 750 | 18.55 | 0.002 | 0.93 |
| 500 | 18.51 | 0.003 | 0.93 |
| 250 | 18.47 | 0.002 | 0.91 |
| 100 | 18.43 | 0.003 | 0.88 |
| 50 | 18.41 | 0.008 | 0.86 |
| 25 | 18.40 | 0.003 | 0.83 |
| 12.5 | 18.40 | 0.003 | 0.79 |
| 6.25 | 18.40 | 0.002 | 0.74 |
| 3.125 | 18.40 | 0.003 | 0.74 |
| 1.563 | 18.40 | 0.001 | 0.72 |
| 0.781 | 18.40 | 0.002 | 0.72 |
| 0.391 | 18.41 | 0.003 | 0.78 |
| 0.195 | 18.41 | 0.003 | 0.85 |
| 0.098 | 18.42 | 0.003 | 0.98 |
| 0.048 | 18.43 | 0.003 | 1.40 |
